# Peer Review of “Mothers’ Knowledge of and Practices Toward Oral Hygiene of Children Aged 5-9 Years in Bangladesh: Cross-Sectional Study”

**DOI:** 10.2196/70142

**Published:** 2025-02-03

**Authors:** Bilkisu Nwankwo

**Affiliations:** 1 Department of Community Medicine, College of Medicine, Kaduna State University, Kaduna, Nigeria

**Keywords:** mother's knowledge and practices, oral hygiene, child oral health, bangladesh

*This is the peer-review report for “Mothers’ Knowledge of and Practices Toward Oral Hygiene of Children Aged 5-9 Years in Bangladesh: Cross-Sectional Study.”*

## Round 1 Review

### Specific Comments

There were a lot of grammatical issues and typographical errors. The manuscript [1] needs to be edited for grammar and syntax. It is also obvious that the manuscript was not proofread adequately.

#### Major Comments

##### Abstract

- A word is missing in the first sentence. Authors should proofread the manuscript.
- Keywords: Dhaka is a more appropriate keyword than Bangladesh.
- Under the Results in the abstract, respondents should be referred to as such and not as samples.

##### Introduction

- The global prevalence of oral diseases was stated, but authors did not capture the prevalence in the study area/country and so have not shown that oral disease is a problem. Even the global prevalence that was stated was only that of dental caries among the seven conditions that make up oral diseases as stated by the authors.
- The objective stated here (last sentence) comes off like the authors are assessing the knowledge and practices of oral hygiene with regard to themselves and not their children as stated in the topic.

##### Methods

- Was it permission that was given by the institutional review board or an ethical clearance?
- This section is quite disorganized. There is a logical flow expected in this section.
- Why was a nonprobability sampling technique (convenient sampling) used for this study? The sampling technique was not explained at all. This will make replicating this study difficult.
- I have an issue with the scoring system and the grading. Is there a reference for it? I particularly have an issue with “moderately average.” It is not a standard term.
- The exclusion criteria are not the opposite of the inclusion criteria as stated by the authors. Exclusion criteria are those already included in the study but that are ineligible for one reason or the other.

##### Results

- In the text above Table 1, authors wrote that most respondents (39.3%) had a monthly family income of “21,000‐40,000 taka per month.” This figure (39.3%) is just over one-third of the respondents and not a majority.
- Table 1: What is the meaning of graduation and above? Is it graduated secondary school or graduated college?
- “Respectively” should be added at the end of the following sentence. “Out of 400 mothers, more than 90% knew the importance of brushing teeth while 82.3% and 80.8% of them knew the recommended frequency and appropriate time for brushing teeth.”

##### Discussion

- The second sentence: the study is not evaluating parent’s knowledge and practices but that of mothers.
- Grammatical errors and missing words

##### Reference List

- Some of the references were not cited correctly. Authors should adhere to the Vancouver referencing style.
